# Lamniform Shark Teeth from the Late Cretaceous of Southernmost South America (Santa Cruz Province, Argentina)

**DOI:** 10.1371/journal.pone.0104800

**Published:** 2014-08-20

**Authors:** Elena R. Schroeter, Victoria M. Egerton, Lucio M. Ibiricu, Kenneth J. Lacovara

**Affiliations:** 1 Department of Biology, Drexel University, Philadelphia, Pennsylvania, United States of America; 2 School of Earth, Atmospheric and Environmental Sciences, University of Manchester, Manchester, United Kingdom; 3 Laboratorio de Paleontología, Centro Nacional Patagónico–Consejo Nacional de Investigaciones Científicas Y Técnicas, Puerto Madryn, Chubut, Argentina; 4 Department of Biodiversity, Earth and Environmental Science, Drexel University, Philadelphia, Pennsylvania, United States of America; University of Oxford, United Kingdom

## Abstract

Here we report multiple lamniform shark teeth recovered from fluvial sediments in the (Campanian-Maastrichtian) Cerro Fortaleza Formation, Santa Cruz Province, Argentina. This small tooth assemblage is compared to various lamniform sharks possessing similar dental morphologies, including *Archaeolamna*, *Cretalamna*, *Dwardius*, *Dallasiella*, and *Cretodus*. Although the teeth share numerous morphological features with the genus *Archaeolamna*, including a developed neck that maintains a relatively consistent width along the base of the crown, the small sample size and incomplete nature of these specimens precludes definitive taxonomic assignment. Regardless, the discovery of selachian teeth unique from those previously described for the region broadens the known diversity of Late Cretaceous South American sharks. Additionally, the discovery of the teeth in fluvial sandstone may indicate a euryhaline paleobiology in the lamniform taxon or taxa represented by this tooth assemblage.

## Introduction

The Cerro Fortaleza Formation (Campanian–Maastrichtian) is located in the Río La Leona Valley in Santa Cruz Province, Argentina. Stratigraphic terminology has been inconsistently applied across this region, and the Cerro Fortaleza Formation is sometimes referred to in previous publications as the Pari Aike, Chorrillo, or Mata Amarilla Formation [1]–[10]. Arbe and Hechem [4] distinguished the Upper Cretaceous fluvial outcrops in the Río Leona Valley as a distinct formation, which we assign to the Cerro Fortaleza Formation. The Cerro Fortaleza Formation is clearly terrestrial and consists predominantly of cross-bedded, friable sandstones interbedded with layers of mudstones and occasional lignitic horizons [1], [2], [4]–[7], [11]–[13]. Based on the presence of paleosols and lignitic horizons, Macellari [12] inferred a humid climate with intervals of high rainfall and a high water table. Avulsion surfaces, histosols [14], carbonaceous root fossils, and silicified wood [15] suggest a low-lying, poorly drained forested terrain.

Recent work in the Cerro Fortaleza Formation has yielded numerous shark teeth, including two nearly complete specimens and multiple fragments. While we cannot entirely discount the possibility of reworking, only a few of the teeth (e.g., MPM-PV 3268) described in this study show evidence of wear (e.g., abrasion, rounding, pitting) or other indicators of significant transportation. This suggests that at least some of the teeth may have been originally deposited in the fluvial environment recorded by the Cerro Fortaleza Formation [4], [6], [7], [12]–[16], indicating they belonged to selachian species capable of tolerating a broad range of salinity. We compare this small tooth assemblage with teeth from various lamniform sharks possessing similar dental morphologies, including *Archaeolamna*, *Cretalamna*, *Dwardius*, *Dallasiella*, and *Cretodus*.

## Materials and Methods

### Locality and Age

**Figure 1:**
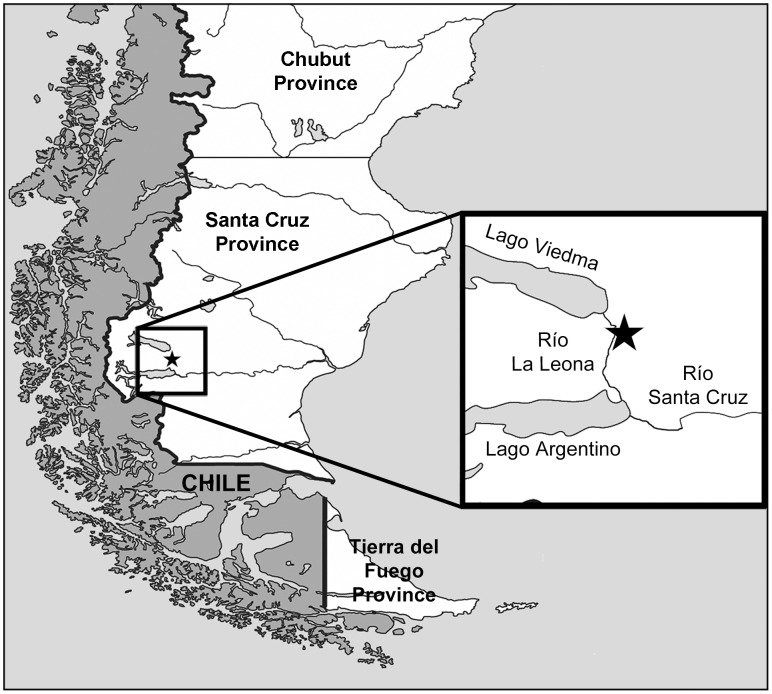
Locality map of discovery site. The star marked on the inset map denotes the location the selachian teeth were recovered from, approximately halfway between the eastern boundaries Lago Viedma and Lago Argentino (adapted from Mapa de la provincia de Santa Cruz, http://es.wikipedia.org/wiki/Archivo).

Selachian teeth were recovered from a site on the southeastern flank of Cerro Fortaleza (49°57′10″S 72° 2′51″W) in Santa Cruz Province, Argentina (fig. 1), from within the Cerro Fortaleza Formation. The teeth were collected from a cross-bedded to planar, medium- to fine-grained sandstone deposited as a channel-fill fluvial facies, bounded by overbank facies with well-developed paleosols. There is a degree of uncertainty with respect to the chronostratigraphic assignment of the Cerro Fortaleza Formation [15]. Although some authors place it in the lower portion of the Upper Cretaceous [14], we agree with those authors assigning it to the Campanian—Maastrichtian stages of the Upper Cretaceous. This assignment is based on biostratigraphic correlation using ammonite and pollen biozones for the underlying Campanian La Anita Formation and the overlying Maastrichtian La Irene Formation, respectively (c.f., [15], [16]).

### Methods

Fieldwork and collection permits were issued by the government of Santa Cruz Province through Museo Padre Molina (Río Gallegos, Argentina), where the specimens are reposited. Teeth were obtained by surface collecting in sediment recently weathered from lithified exposures of fluvial sandstone and overbank shale facies of the Cerro Fortaleza Formation over an area of approximately 500 m^2^. The site is located on a bald mountaintop that allows for the accumulation of very little unconsolidated sediment, but also precludes the possibility of modern transportation in from other locations. The limited amount of weathered material available did not permit sampling of the site via screen washing.

For the description, we follow the tooth terminology of Shimada [17]. The dental measurements (e.g. tooth height, crown height, mesial cutting edge length, distal cutting edge length) follow Shimada [18], with the addition of distal inclination (DI), sensu Cook [19].

### Material

**Figure 2:**
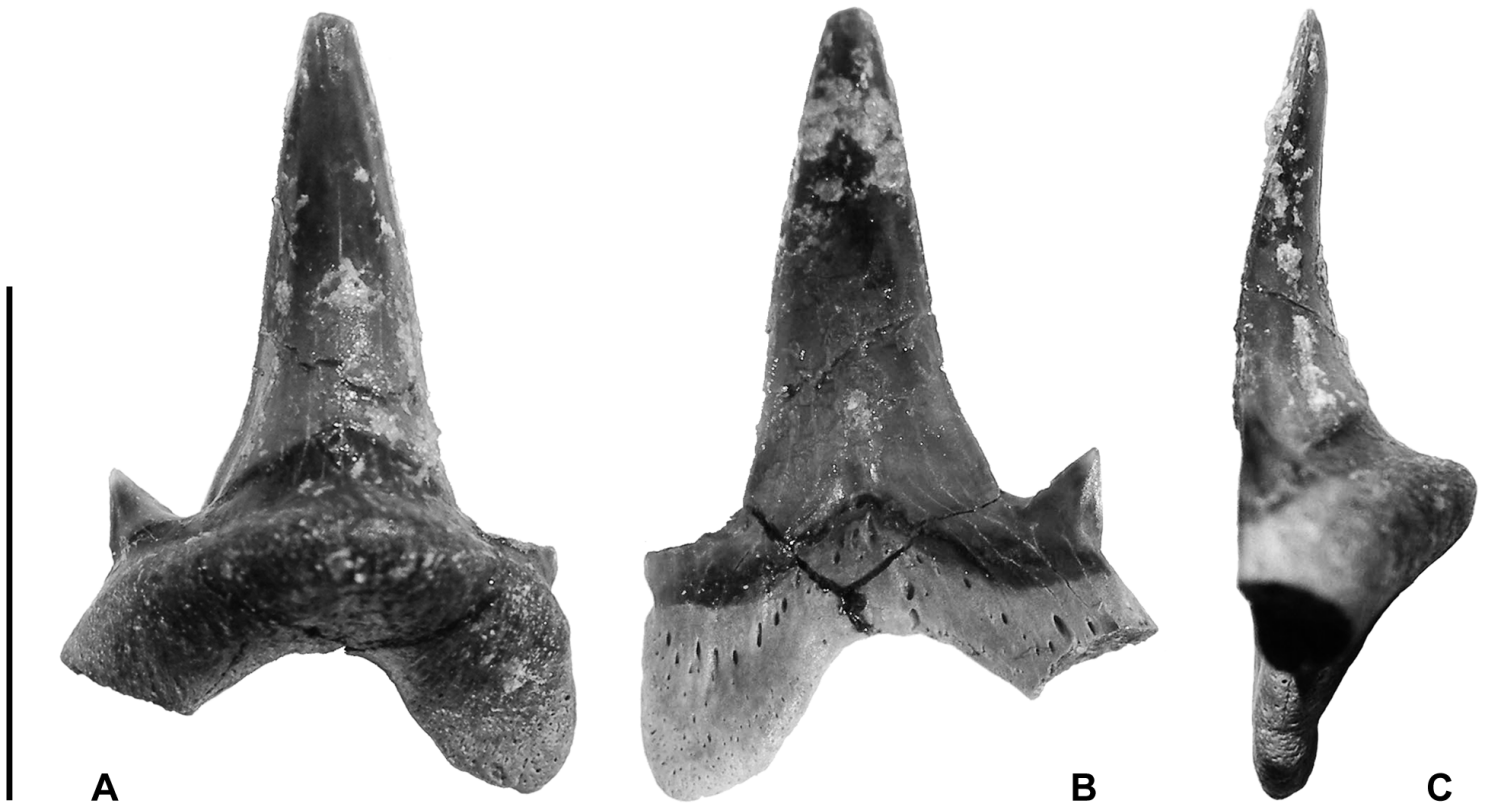
MPM-PV 3267. Lamniform tooth in (A) lingual, (B) labial, and (C) lateral view. Note the slenderness of the principle cusp, the well-pronounced lingual protuberance lacking a nutrient groove, and the developed neck that maintains a constant width across all cusps. Scale bar equals 1 cm.

**Figure 3:**
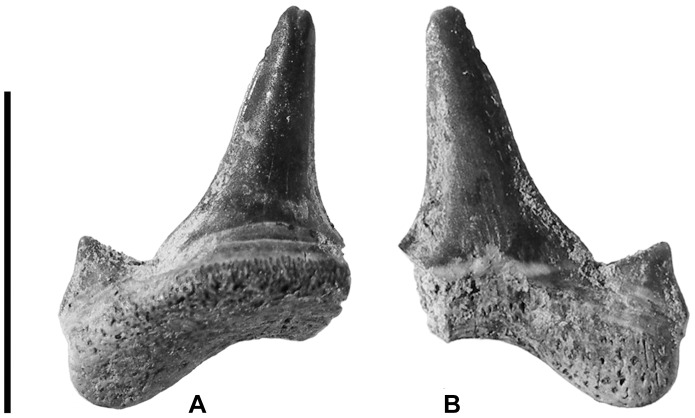
MPM-PV 3268. Lamniform tooth in (A) lingual, and (B) labial view. Note distinct narrowing in the apical two-thirds of principle cusp. Scale bar equals 1 cm.

**Figure 4:**
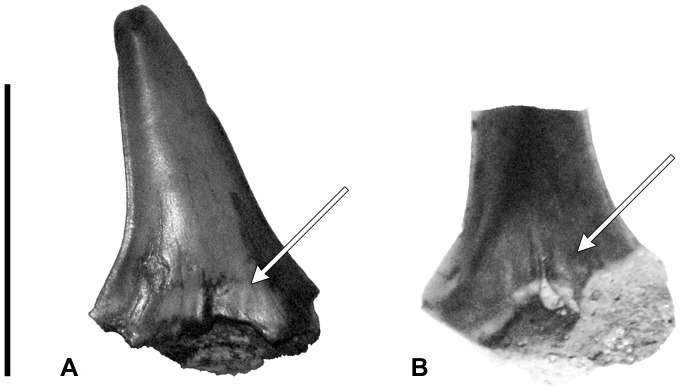
Lamniform crown fragments in labial view. (A) MPM-PV 3269, (B) MPM-PV 3270. Arrows point to longitudinal ridges (enameloid folding) at the base of the crown. Scale bar equals 1 cm.

**Figure 5:**
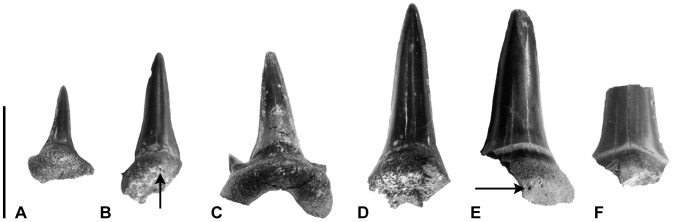
A selection of recovered tooth fragments showing size range and overall symmetry of crowns. (A) MPM-PV 3271, (B) MPM-PV 3272, (C) MPM-PV 3267, (D) MPM-PV 3273, (E) MPM-PV 3274, (F) MPM-PV 3275. All fragments are in lingual view. Arrows point to small nutrient foramina. Note that in all specimens, the lingual neck maintains a relatively equal width across the base of the crown. Scale bar equals 1 cm.

Two nearly complete specimens (MPM-PV 3267 and MPM-PV 3268) were recovered from the Cerro Fortaleza Formation, Santa Cruz Province, Argentina. MPM-PV 3267 is complete, except for the tip of one root lobe and the tip of the opposing lateral cusplet (fig. 2). Though the apical portion of this lateral cusplet is missing, the preserved base and breakage surface show that it was present and was singular. MPM-PV 3268 is missing the entire distal root lobe, including the (presumed) attached cusplet (fig. 3). Additional fragments (MPM-PV 3269–MPM-PV 3275, fig. 4–5) possessing morphology consistent with the two complete specimens plus additional informative characters were also recovered from the same horizon.

### Institutional Abbreviations

MPM: Museo Padre Molina, Río Gallegos, Santa Cruz.

### Associated Fauna

Surface collecting at this locality produced additional material, including fragments of dinosaur bones, dipnoan teeth, testudine shell fragments and semionotiform/lepisoteid scales. Though effort was made to collect all exposed microfossils, the selachian teeth described in this study were the only chondrichthyan teeth present.

## Results

### Description

#### MPM-PV 3267

This specimen (fig. 2, A–C) bears a tall, erect central cusp that is smooth on both the labial and lingual faces with no apparent longitudinal ridges ( =  enamel folding, vertical folds). The flat labial face and the strongly convex lingual face meet at a non-serrated cutting edge that curves in a weak sigmoid shape in lateral view. This cutting edge runs continuously over a pair of non-serrated, triangular lateral cusplets that are slightly divergent relative to the central cusp. The connection between all three cusps is only apparent on the labial face of the tooth. On the lingual face, there is a distinctive neck at the base of the crown that stretches over both the central cusp and lateral cusplets. The root is bilobate, and the root lobe tip that is preserved is rounded. A deep basal concavity indents the root in a U-shape, and the basal edge of each root lobe forms an internal angle of approximately 90°. The lingual protuberance is pronounced and somewhat restricted medially, tapering towards the basal ends of the root lobes. There is no nutrient groove or nutrient foramina.

#### MPM-PV 3268

This specimen (fig. 3, A–B) bears all the characteristics described for MPM-PV 3267 above, with the following exception: the U-shaped basal concavity is broader and more shallow than in MPM-PV 3267. The lingual protuberance is less pronounced and appears to bear multiple nutrient foramina, though these features may be an erosional artifact. The central cusp displays a slight but distinct distal inclination and significantly narrows in the apical two-thirds of its length.

#### Additional Fragments

Nine fragments were recovered from the locality. These include cusps and partial cusps with portions of the root attached. Complete root lobes and lateral cusplets have not been preserved on any of these specimens. Although the fragmentary nature of the teeth do not allow for a confident diagnosis on an individual basis, the morphological features of the preserved crown and root portions completely overlap with those described above for MPM-PV 3267 and MPM-PV 3268, and also provide additional informative characters. Fragments MPM-PV 3269 and MPM-PV 3270 display weak enamel folding at the base of the crown on the labial face (fig. 4, A–B), but not on the lingual face. Fragments MPM-PV 3272 (fig. 5, B) and MPM-PV 3274 (fig. 5, E) bear a small, singular nutrient foramen on the lingual protuberance. In neither case does the foramen lie in a nutrient groove. All crown fragments bear a neck that maintains a relatively constant width across the entire base. Refer to Table 1 for measurement data of all teeth and tooth fragments.

**Table 1:** Dental measurements for recovered tooth fragments.

Specimen	TH	CH	MCL	DCL	DI
MPM-PV 3267	15.47	11.39	10.26	10.04	0.98
MPM-PV 3268	12.53	10.74	9.36	∼8.54	0.91
MPM-PV 3269	>12.79	—	—	—	—
MPM-PV 3270	—	—	—	—	—
MPM-PV 3271	>8.70	8.11	—	—	—
MPM-PV 3272	>13.53	—	—	—	—
MPM-PV 3273	>18.42	—	—	—	—
MPM-PV 3274	>17.85	—	—	—	—
MPM-PV 3275	—	—	—	—	—

All measurements are in millimeters. “>” indicates that broken surfaces preclude full measurement; given value significantly less than for the complete tooth. Abbreviations: TH, tooth height; CH, crown height; MCL, mesial cutting edge length; DCL, distal cutting edge length; DI, distal inclination.

### Comparisons

#### *Archaeolamna*

*Archaeolamna* is a widely distributed Cretaceous euselachian genus ranging from the Albian to the Maastrichtian [20], and includes the species *A. kopingensis*, *A.* ex. gr. *A. kopingensis*, *A.* cf. *A. kopingensis*, *A.* aff. *kopingensis*, *A. kopingensis judithensis*, and *A. haigi*. Though described as having a “virtually global” distribution [21], *Archaeolamna* has been reported primarily from the Northern Hemisphere, with occurrences on four continents: Europe, including England (*Lamna arcuata* and *Plicatolamna arcuata*) [22], Belgium [23], France [24], [25], and Sweden (*Odontaspis kopigensis* [26], [27]; Asia, including Russia (*Pseudoisurus tomosus*) [20] and Kazakhstan [28], North America, including Canada [21], [29]–[31] and the United States [19], [32]–[37]; and Australia [20], [38]. Specimens MPM-PV 3267–MPM-PV 3275 share the following characteristics with *Archaeolamna kopingensis*: (1) tall, triangular principle cusps with strongly convex lingual faces and flat or slightly convex labial faces, flanked by a single pair of lateral cusplets; (2) a continuous, non-serrated cutting edge that runs across the principle cusp and cusplets; (3) a bilobate root with a deeply indented basal concavity and pronounced lingual protuberance that bears no nutrient groove, and variably one nutrient foramen; and (4) a developed neck that maintains a relatively consistent width along the base of the crown [20], [21], [27], [38], [39].

Unlike the teeth of *A. kopigensis*, the root/crown boundary is strongly indented on the labial face of the Cerro Fortaleza specimens (per. comm, M. Siversson, 2014). The principle cusp also appears to be more elongate and slender than the robust, broad crown commonly seen in *A. kopingensis*, and is more similar to *A. haigi* in this regard [38]. Underwood and Cumbaa [21] noted that there was a robust form and a gracile form present in a Cenomanian assemblage of *Archaeolamna* ex. gr. *kopingensis* from Saskatchewan, and suggested that this might represent a sexually dimorphic characteristic. The specimens described here represent multiple individuals, all possessing a more gracile morphology.

The Cerro Fortaleza *Archaeolamna* are differentiated from the *A. haigi* by the lack of enamel folding on the lingual face of the crown. Additionally, the labial crown face of *A. haigi* anterior teeth are typically concave, whereas our specimens have a flat to slightly convex labial crown face [19], [20].

#### *Cretalamna*

A recent reevaluation of *Cretalamna* has expanded the genus to include eight species, including *C. appendiculata, C. borealis*, and six undescribed species [40]. The root lobes of species within *Cretalamna* are typically quadrangular in shape, with a flattened ventral margin in lateral profiles [18], [39]–[41]; this feature is not observed in any of the present specimens with intact roots. Additionally, two of the fragments, MPM-PV 3269 and MPM-PV 3270, possess weak enamel folding along the labial side of the crown base. Within *Cretalamna*, such longitudinal ridges are only present in two species [40], and then only in lateroposterior teeth, which have more flattened root lobes and more recurved principle cusps than observed in any of the Cerro Fortaleza specimens.

#### *Dwardius*

The Cerro Fortaleza teeth are differentiated from *Dwardius* by the relatively constant width of the neck across the base of the central cusp and lateral cusplets (see fig. 5); *Dwardius* crowns bear a neck that tapers laterally, becoming significantly thinner beneath the lateral cusplets [42]. Additionally, the central cusp of the teeth in this study narrow in the apical two-thirds (exemplified in the Cerro Fortaleza specimens most clearly by MPM-PV 3272, fig. 5B), a feature not present in *Dwardius* [19], [42].

#### *Dallasiella*

These specimens are differentiated from *Dallasiella willistoni* by the absence of a nutrient groove on any of the preserved root fragments. Whereas the lingual protuberance of *D. willistoni* specimens bear a short nutrient groove housing a large foramen [31], all of the Cerro Fortaleza specimens lack a nutrient groove, and only a few (e.g. MPM-PV 3272 and MPM-PV 3274, figs. 5B and 5F) possess a small nutrient foramen.

#### *Cretodus*

Our specimens are differentiated from *Cretodus* by the absence of longitudinal enamel folds on the lingual face of all specimens. Additionally the distinct longitudinal ridges at the base of the crown on both the labial and lingual faces is a characteristic feature for the genus is absent [39], [43].

## Discussion

The overall symmetry and proportions of the Cerro Fortaleza lamniform tooth assemblage across a size range (see Table 1 and fig. 5) suggests that most of the specimens recovered are anteriorly situated teeth. Generally, lamniform principle cusps are distally directed in lateral tooth files in genera such as *Archaeolamna* [21] and *Cretalamna* [18], [40], especially in the upper jaw. In contrast, the majority of tooth crowns collected from Cerro Fortaleza are erect and nearly symmetrical (fig. 5). MPM-PV 3269 represents the most asymmetrical tooth crown recovered (see fig. 4), and even the smallest tooth, MPM-PV 3271 (>8.7 mm), maintains a nearly symmetrical cusp. Because the locality of discovery did not allow for more thorough collection techniques than surface picking (e.g., screenwashing), it is likely that these specimens were subject to a collection bias towards larger teeth. Thus, the exclusive collection of teeth towards the anterior portion of the jaw suggests the anterior teeth were enlarged relative to their lateral counterparts; otherwise, teeth from both jaw sections should have been recoverable by surface picking (see [40]). In any case, the lack of lateral teeth in this assemblage, as well as any fully completed specimens, limits the diagnostic features available for analysis and makes definitive identification of these teeth, even to a single species, challenging.

South America has a rich record of fossil lamniform sharks (e.g., *Carcharocles*, *Carcharodon*, *Carcharoides*, *Carcharias*, *Cretalamna*, *Isurus*, *Odontaspis*, *Scapanorhynchus*, *Serratolamna*, and *Squalicorax*) [41], [44], [45]. Though further fieldwork to produce a larger sample set with a reduced collecting bias towards large teeth is necessary for a definitive assignment of these specimens, the discovery of teeth unique from those previously described for the region broadens the known diversity of Late Cretaceous South American sharks.

The recovery of these teeth from what is widely accepted to be a fluvial depositional environment [4], [6], [7], [12]–[16] suggests the taxon or taxa represented by this assemblage may have been able to tolerate a broad range of salinity, similar to the modern bull shark *Carcharhinus leucas* [46], [47]. This interpretation is additionally supported by the exclusively terrestrial and freshwater associated fauna recovered at the locality. Further, recent studies suggest that euryhalinity may be more common in extant marine elasmobranchs than previously reported [48], a phenomena that may apply to extinct forms as well. However, possible wear on the roots of at least one specimen, MPM-PV 3268 (fig. 3), leaves open the possibility that some of these teeth may have been transported or reworked from marine sediments. Future discoveries and sedimentological work in the region is needed to resolve the taxonomic affinity and euryhalinity of this enigmatic tooth assemblage.
